# ASO Author Reflections: Neoadjuvant Chemotherapy Switch: Optimizing Neoadjuvant Treatment Sequencing in Pancreas Cancer

**DOI:** 10.1245/s10434-021-11019-5

**Published:** 2021-10-27

**Authors:** Roberto Alva-Ruiz, Mark J. Truty

**Affiliations:** Division of Hepatobiliary and Pancreas Surgery, Mayo Clinic, Rochester, MN USA

## Past

Neoadjuvant chemotherapy (NAC) is considered for patients with borderline resectable/locally advanced (BR/LA) pancreatic adenocarcinoma (PDAC). A chemotherapeutic regimen that is both tolerable and demonstrably effective is of utmost importance as oncologic outcomes in these higher risk patients highly correlate with subsequent pathologic treatment responses.^1^ However, there are little data on the optimal treatment strategy in patients who do not objectively respond to or develop treatment-limiting toxicities with first-line (FL) NAC.^2–4^ In recent years, our center has adopted a ‘chemotherapy switch’ (CS) strategy for such patients. In our manuscript entitled *‘Neoadjuvant Chemotherapy Switch in Borderline Resectable/Locally Advanced Pancreatic Cancer’* we report on our cumulative high-volume institutional experience with CS, including frequency, indications, and outcomes of patients who require CS after initial FL NAC prior to curative-intent surgery.^5^

## Present

We were able to demonstrate that a substantial proportion (30%) of patients required a chemotherapeutic switch after initial FL NAC due to the lack of objective treatment responses and/or chemotherapeutic toxicities. Of those undergoing CS, a majority were able to proceed to curative-intent surgery after subsequent CS. We found no major preoperative or perioperative differences between those undergoing resection after FL NAC compared with those who required CS. Importantly, there were no differences in pathologic or survival outcomes between cohorts, suggesting that such a CS strategy does not incur an oncologic detriment and can potentially be used to salvage those patients in whom FL chemotherapy is not tolerable or is ineffective.^5^

## Future

The presented results contribute to our overall understanding of appropriate neoadjuvant treatment sequencing and highlight the continued evolution of what constitutes an optimal treatment strategy for patients with anatomically borderline resectable or locally advanced PDAC. Based on these results, we suggest that CS be considered for all patients with initial suboptimal responses or in those who develop treatment-limiting toxicities to FL as a significantly important neoadjuvant strategy prior to consideration of surgical resection in order to improve outcomes for these patients. We anticipate the use of such a strategy combined with evolving NAC response metrics will improve the optimal selection of patients who could potentially benefit from curative-intent surgical resection.
